# Deep learning-based virtual H& E staining from label-free autofluorescence lifetime images

**DOI:** 10.1038/s44303-024-00021-7

**Published:** 2024-06-28

**Authors:** Qiang Wang, Ahsan R. Akram, David A. Dorward, Sophie Talas, Basil Monks, Chee Thum, James R. Hopgood, Malihe Javidi, Marta Vallejo

**Affiliations:** 1 Centre for Inflammation Research, Institute of Regeneration and Repair, The University of Edinburgh, Edinburgh, UK; 2 Translational Healthcare Technologies Group, Centre for Inflammation Research, Institute of Regeneration and Repair, The University of Edinburgh, Edinburgh, UK; 3 Department of Pathology, Royal Infirmary of Edinburgh, Edinburgh, UK; 4 School of Engineering, The University of Edinburgh, Edinburgh, UK; 5 School of Mathematical and Computer Sciences, Heriot-Watt University, Edinburgh, UK; 6 Department of Computer Engineering, Quchan University of Technology, Quchan, Iran

**Keywords:** Engineering, Cancer imaging

## Abstract

Label-free autofluorescence lifetime is a unique feature of the inherent fluorescence signals emitted by natural fluorophores in biological samples. Fluorescence lifetime imaging microscopy (FLIM) can capture these signals enabling comprehensive analyses of biological samples. Despite the fundamental importance and wide application of FLIM in biomedical and clinical sciences, existing methods for analysing FLIM images often struggle to provide rapid and precise interpretations without reliable references, such as histology images, which are usually unavailable alongside FLIM images. To address this issue, we propose a deep learning (DL)-based approach for generating virtual Hematoxylin and Eosin (H&E) staining. By combining an advanced DL model with a contemporary image quality metric, we can generate clinical-grade virtual H&E-stained images from label-free FLIM images acquired on unstained tissue samples. Our experiments also show that the inclusion of lifetime information, an extra dimension beyond intensity, results in more accurate reconstructions of virtual staining when compared to using intensity-only images. This advancement allows for the instant and accurate interpretation of FLIM images at the cellular level without the complexities associated with co-registering FLIM and histology images. Consequently, we are able to identify distinct lifetime signatures of seven different cell types commonly found in the tumour microenvironment, opening up new opportunities towards biomarker-free tissue histology using FLIM across multiple cancer types.

## Introduction

Fluorescence lifetime is characterised by a decay from the excited state to the ground state, which is independent of its intensity but extremely sensitive to the surrounding bio-environment. Fluorescence lifetime imaging microscopy (FLIM) has been shown to provide valuable information about the underlying metabolic state, pathological conditions, and constitution of biological samples by analysing endogenous fluorescence^1,2^. By measuring the fluorescence lifetime of fluorescent molecules within a sample, FLIM can provide insights into various microenvironmental factors, including pH, ion concentration, and molecular interactions. Due to lifetime contrast, this has led to various applications in biology and medicine, such as cancer detection and diagnosis^1,3^, cellular phenotyping^4,5^, pH calculation of the secretory pathway^6^, and the monitoring of drug delivery^7^.

Conventionally, quantitative analysis of lifetime contrast is prevailed by statistical methods, such as histogram of FLIM images or phasor characterisation^2,4,8^, with the assistance of reference data, e.g., histological images. Due to the nature of statistical analysis, most current approaches can only reveal averaged lifetime of dominant components in FLIM images, rather than cellular-level information. Although a few papers^9,10^ have addressed this by co-registering FLIM and histology images together, there are still outstanding challenges associating with this co-registration, such as underlying structure change caused by tissue preparation^11^. In addition, such reference data is not always available alongside FLIM images, and therefore, unable to provide instant interpretation immediately after FLIM imaging procedures.

The explosive development of deep learning (DL) technologies is transforming conventional biomedical imaging analysis^12,13^, leading to automated processing that could surpass human capabilities. One of the beneficial fields is virtual histological staining from microscopic images acquired by various imaging modalities^14^ using DL models, among which U-Net^15^-based Generative Adversarial Networks (GANs)^16^ are prominent. Rivenson et al.^17^ demonstrated the effectiveness of translating label-free autofluorescence intensity images to multiple histology staining for different organs using a supervised GAN with an extra constraint of total variation. Li et al.^18^ synthesised bright-field images on unstained carotid artery samples to Haematoxylin and Eosin (H&E), Picrosirius Red, and Orcein stain using the pix2pix GAN^19^. Borhani et al.^20^ applied a custom DL model to generate virtual H&E stain from a combination of 16-channel two-photon excited fluorescence images with 1-channel lifetime images collected by multi-modal microscopy on unstained tissue. Cao et al. applied an unsupervised GAN model, namely CycleGAN^21^, to transform ultraviolet photoacoustic microscopy to virtual H&E staining^22^. Other examples include transferring H&E to virtual IHC (immunohistochemistry) stain^23^ and IHC to virtual multiplex immunofluorescence^24^.

In this study, we will explore the use of DL techniques to generate virtual H&E staining from label-free FLIM images. Specifically, we will employ the supervised DL model, pix2pix GAN^19^, along with the advanced image quality metric, Deep Image Structure and Texture Similarity (DISTS)^25^, to synthesise H&E-stained histology images. By doing so, we aim to demonstrate the potential of this technique in enabling rapid and precise interpretation at the cellular level of FLIM images, which could allow for the identification of label-free autofluorescence lifetime signatures for various types of tumour cells. Additionally, we will compare the effectiveness of different input data formats, such as intensity and weighted lifetime images, to showcase the benefits of using FLIM for the synthesis of virtual H&E staining. Meanwhile, we will also evaluate our method for multiple cancer types to demonstrate the advantages of FLIM-based virtual H&E staining.

## Results

### Virtual H&E staining from FLIM

**Fig. 1:**
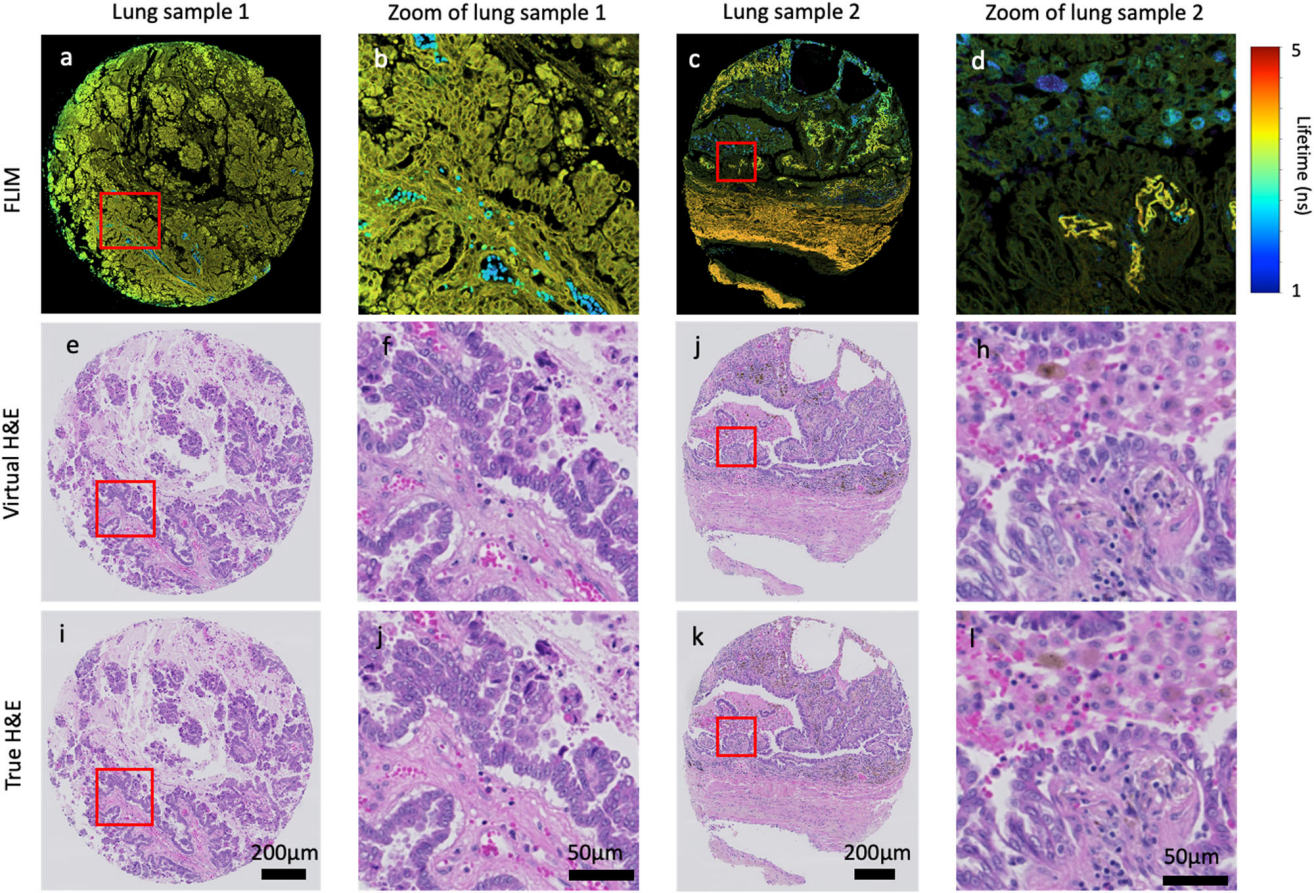
Virtual H& E staining from label-free FLIM images of non-small cell lung cancers. (**a**) and (**c**) are the false-colour FLIM images inputted into the DL model, where (**b**) and (**d**) are the corresponding patches within the red rectangle, respectively. Corresponding virtual H& E images are shown (**e**–**h**), along with true H& E images (**i**–**l**).

Figure 1 summarises examples of the virtual H&E staining from label-free FLIM images using an independent dataset collected on two TMA (tissue microarray) cores of lung cancer which were not utilised in training, where one contains relatively few distinct cell populations (Fig. 1a) while the other is a more complex mixture of cell types (Fig. 1c). Both are visualised as intensity-weighted false-colour lifetime images (see the section “Comparison of different image formats” for the details).

The results demonstrate that the method described in the section “Methods” is able to reconstruct various cellular components in the lung tissue samples from FLIM images to match the corresponding H&E-stained brightfield images. Specifically, the method proves its effectiveness in faithfully reproducing the morphological and textural attributes of a wide range of cell types present within the lung TME (tumour micro-environment), including tumour cells, stromal components (such as fibroblast’s) and inflammatory cells, as well as red blood cells. Each of these was confirmed with the True HE (Fig. 1i–l).

While the DL model was trained using input patches sized at 256 × 256, it is important to note that these models can accommodate input of various sizes during the inference stage. In addition, H&E-stained images were downsampled to match the pixel size of FLIM images, where the pixel size was increase from 0.22 *μ*m to 0.455 *μ*m using bicubic interpolation.

### Blind evaluation of virtual staining quality

In pathology laboratories, the visual inspection of histological images by experienced pathologists is an integral step in current clinical practice for reaching diagnostic decisions. Similar to refs. ^17,26^, a blind evaluation was conducted by three experienced pathologists to assess the subjective quality of virtual H&E staining. 12 pairs of true and virtual H&E-stained images were prepared using lung cancer tissues, with all images anonymised. To comprehensively evaluate the quality of virtually stained images, the pathologists examined each image in four key aspects, including nuclei detail via Haematoxylin stain, cytoplasm detail via Eosin stain, overall staining quality, and quality for diagnostic decision-making. For each dimension, pathologists assigned scores based on predetermined criteria, ranging from Unacceptable (1) to Acceptable (2), Excellent (3), and Perfect (4). This meticulous evaluation process offers valuable insights into the efficacy and dependability of virtual H&E staining compared to conventional staining methodologies.

**Fig. 2:**
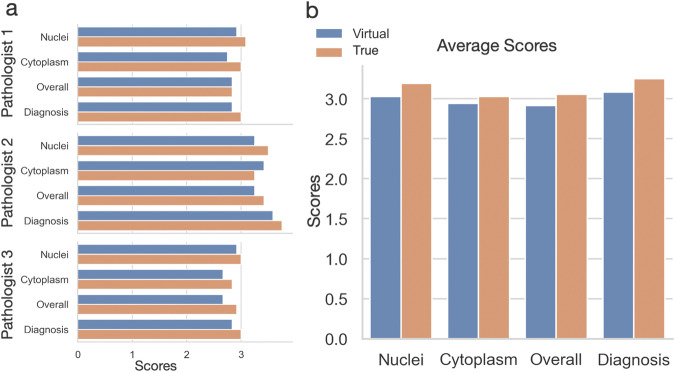
Blind evaluation of virtual and true H& E stained lung cancer cores. **a** Subjective quantification of true and virtual H& E staining of 12 TMA cores by three pathologists. **b** Overall quantification by the inter-observer scores of true and virtual histological images. Each score combines the evaluation of nuclear and cytoplasmic detail, overall staining quality, and diagnostic confidence based on staining quality, quantified using a range of 1 (unacceptable) to 4 (perfect).

Figure 2 depicts the mean scores provided by each pathologist on four image quality metrics. In general, the average scores shown in Fig. 2b demonstrate the outstanding quality of virtual histological images on all metrics, where only marginal differences were found between real and virtual images. Although each pathologist rated the images differently (Fig. 2a), their outcomes are consistent on the differences between true and virtual images. For example, pathologist 1 ranked both stains are above excellent, while pathologist 3 considered they are between acceptable (2) and excellent (3). Supplementary Table 1 presents the detailed ranking on each lung tissue sample. An interesting result is on sample 6 (lung sample 2 in Fig. 1), where all pathologists voted the virtual image over the true one on all four metrics. In summary, the subjective evaluation demonstrated a high degree of agreement between the techniques, where the pathologists were able to examine histological features on virtual staining and reach the same level of diagnostic decision-making.

### Vitual H&E staining on various tissue samples

**Fig. 3:**
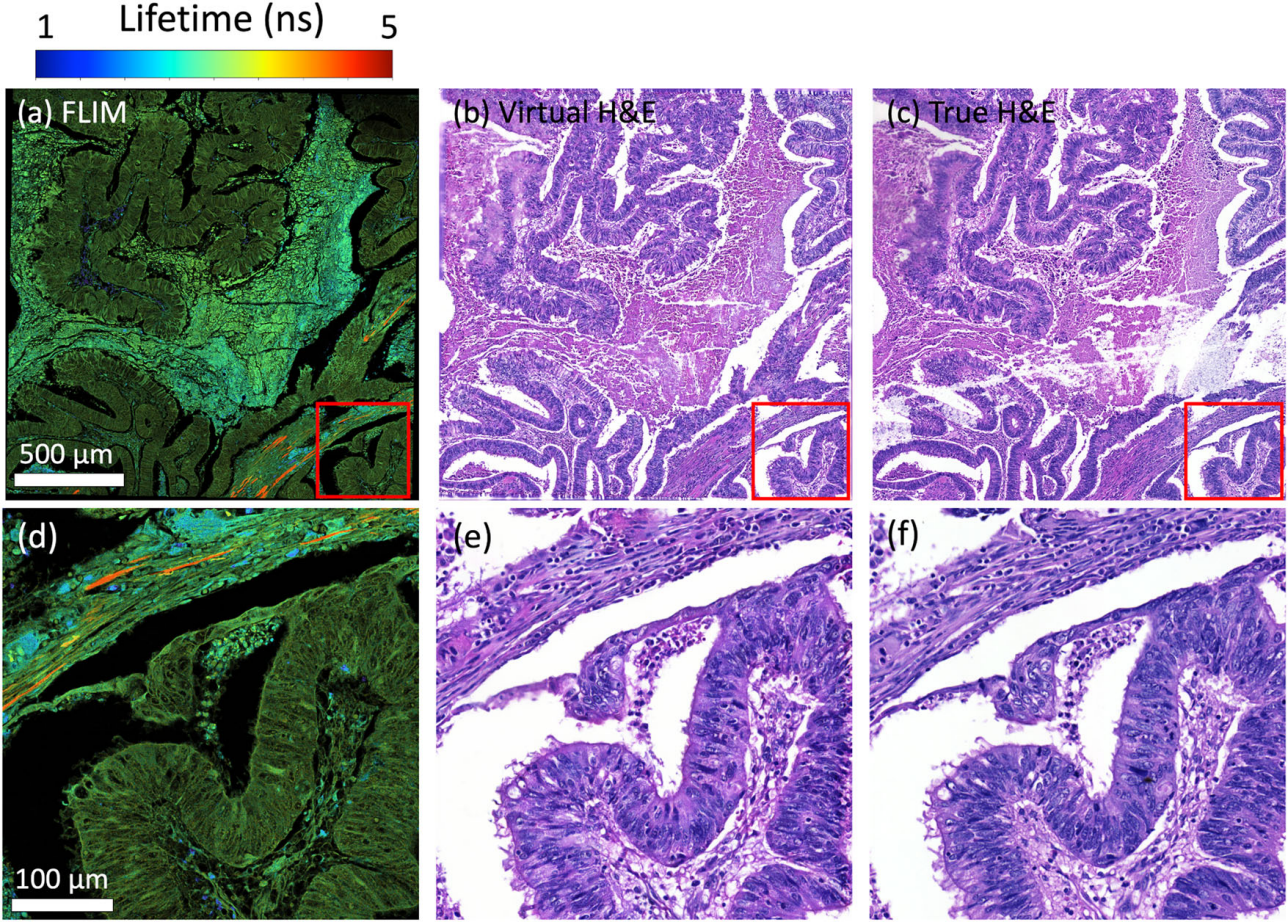
Virtual H& E staining from FLIM on colorectal adenocarcinoma. (**a**) and (**d**) are intensity-weighted FLIM images. (**b**) and (**e**) are the virtually stained images. (**c**) and (**f**) are the true H& E stain as the reference.

**Fig. 4:**
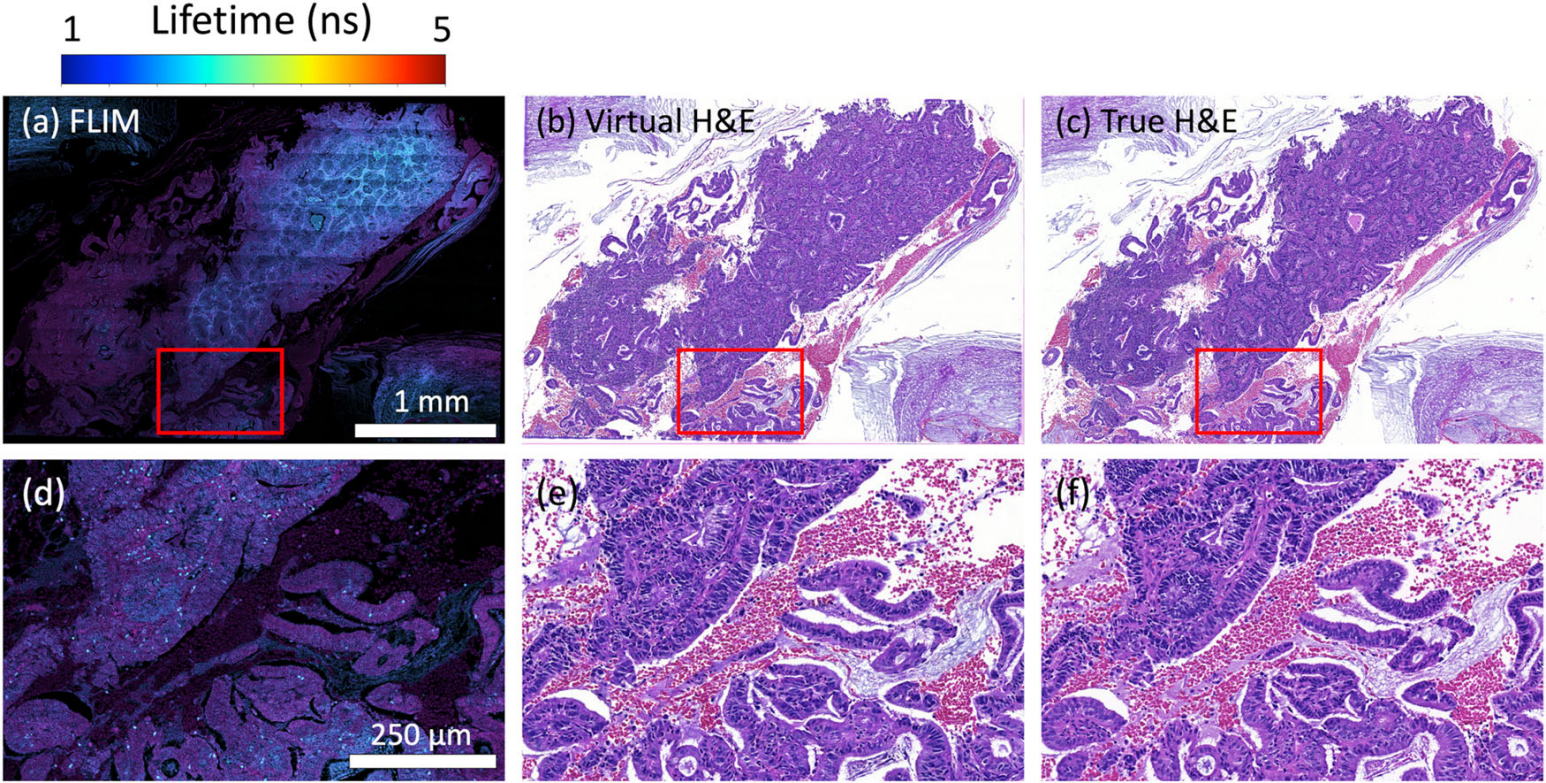
Virtual H& E staining from FLIM on endometrial adenocarcinoma. (**a**) and (**d**) are intensity-weighted FLIM images. (**b**) and (**e**) are the virtually stained images. (**c**) and (**f**) are the true H& E stain as the reference.

In addition to lung tissue samples, we expanded our experiments to include primary colorectal and endometrial cancers, and the results are presented in Figs. 3,4 for colorectal and endometrial adenocarcinomas, respectively. Similar to the results shown in the section “Virtual H&E staining from FLIM”, the virtual images (Fig. 3b, e for colorectal cancer and Fig. 4b, e for endometrial cancer) exhibit significant consistency with the real ones (Fig. 3c, f for colorectal cancer and Fig. 4c, f for endometrial cancer), where most celluar details are accurately reconstructed, which are suitable for both visual and quantitative analysis. A notable phenomenon is observed in the FLIM image of endometrial cancer, where the fluorescence lifetime appears dramatically shorter compared to other samples, resulting in a visually dimmer appearance. However, it’s important to note that this shorter lifetime does not affect the virtual result obtained through our method.

**Fig. 5:**
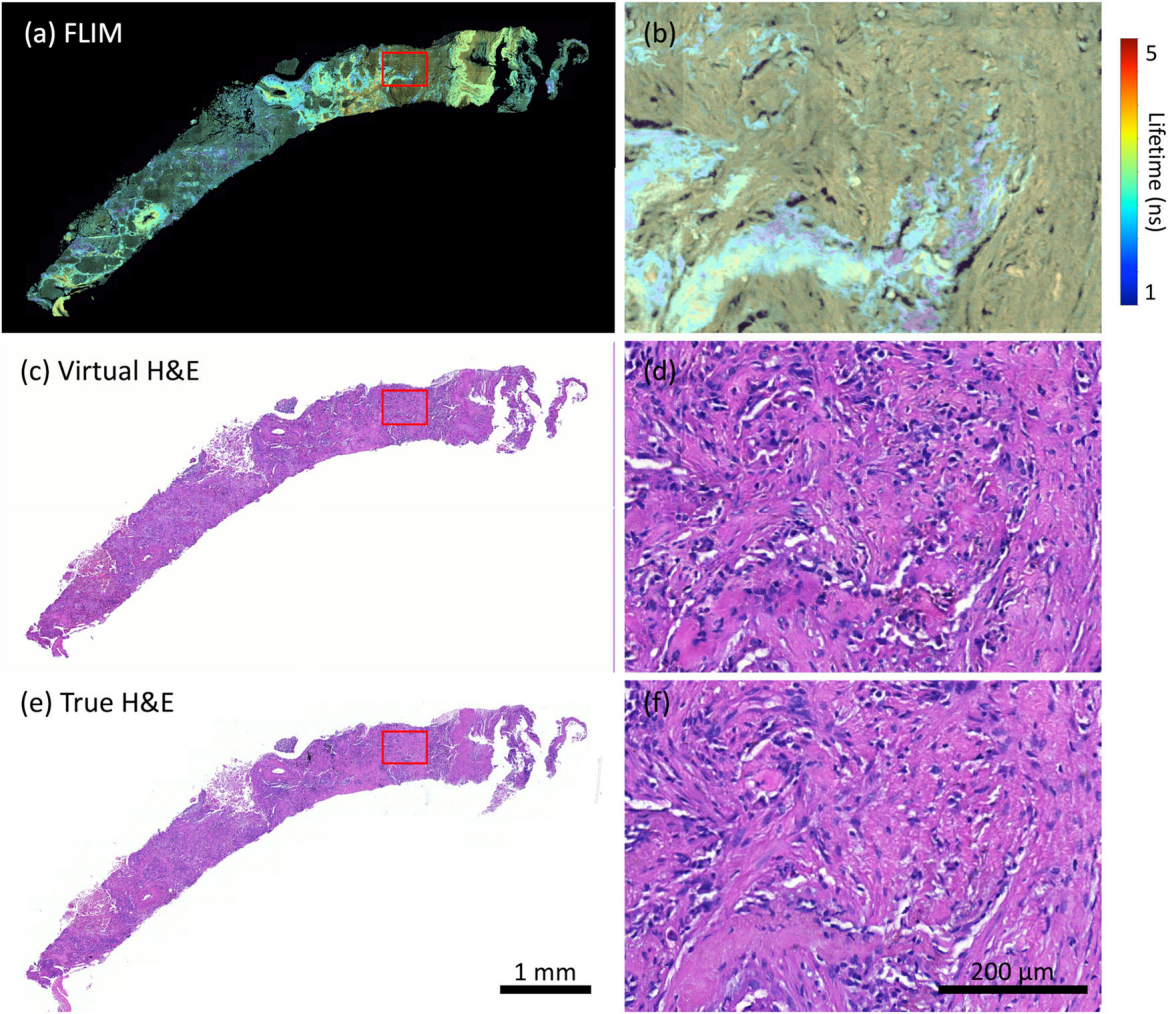
Virtual H& E staining on an Formalin-Fixed Paraffin-Embedded lung biopsy sample. (**a**) and **b** are FLIM images. (**c**) and (**d**) are the virtually stained images. (**e**) and (**f**) are the true H& E stain as the reference.

We also expanded our experiments to include Formalin-Fixed Paraffin-Embedded (FFPE) lung biopsies. Figure 5 summarise the results of an FFPE lung biopsy. The virtually stained image (Fig. 5d) of the lung biopsy exhibits consistency with the real one (Fig. 5f), revealing cellular details within the area. Despite the performance of our method on the biopsy is not as remarkable as demonstrated on TMA slides in the section “Virtual H&E staining from FLIM”, the virtual image does not compromise clinical decision-making.

### Lifetime signatures of various cell types in lung tissue

**Fig. 6:**
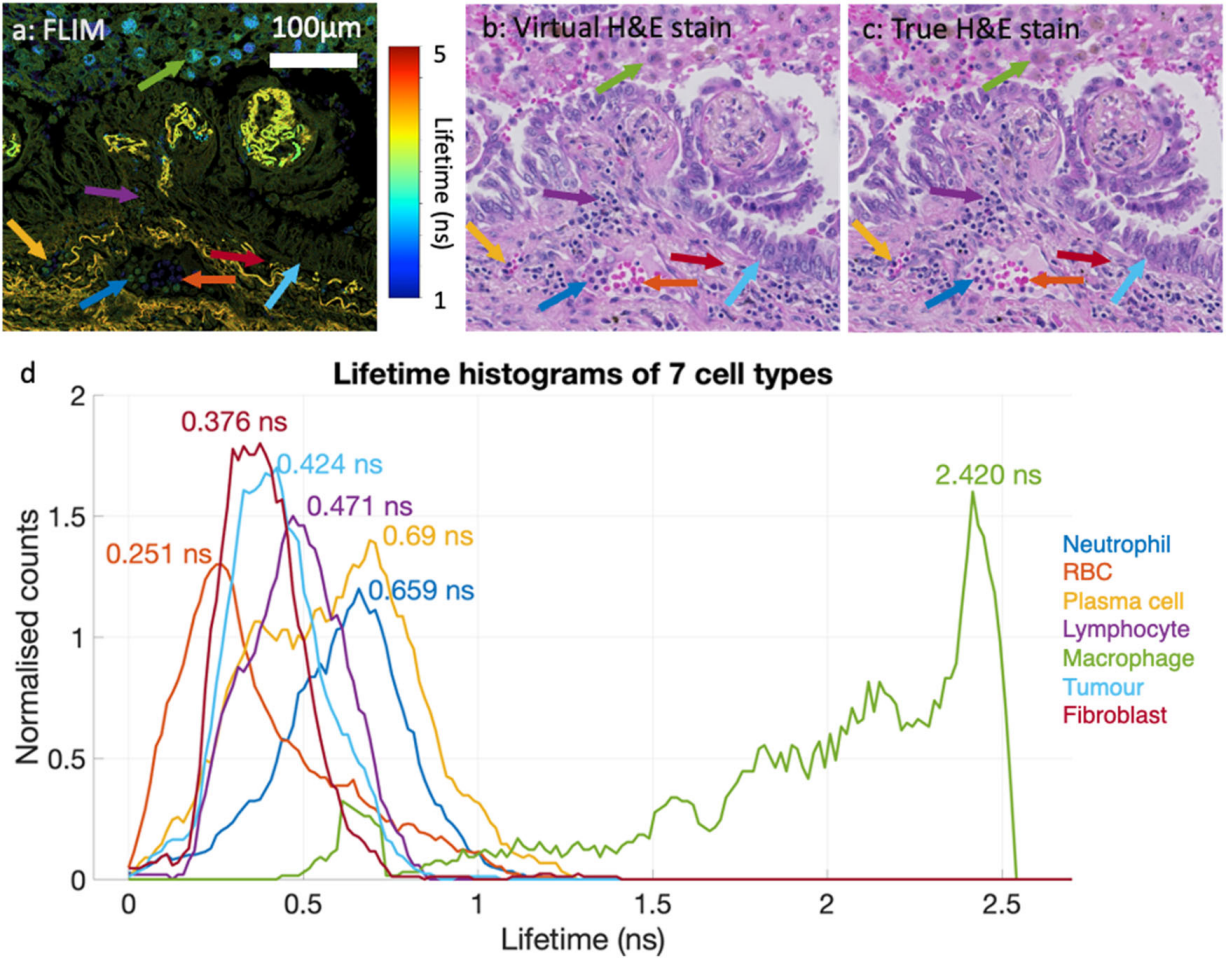
Lifetime signatures of 7 cellular components in lung cancerous tissue, including tumours, lymphocytes, plasma cells, macrophages, neutrophils, fibroblasts, and red blood cells (RBC). DL-generated virtual H& E (**b**) has the capability to reconstruct those cell types from the FLIM image (**a**), enabling precise and timely identification of lifetime signatures of cellular components in the tissue. True H& E image is also presented for comparison. (**d**) demonstrates that 7 cellular components annotated have distinct lifetime values.

By leveraging virtual H&E staining, we can directly map cellular components annotated by pathologists to FLIM images, enabling the identification of lifetime signatures of diverse cell types, without the need for conventional staining and co-registration processing procedures. Figure 6 depicts an example of label-free autofluorescence lifetime signatures of 7 different cell types, where the lifetime is determined by the peak values in the histogram of pixel lifetime values within the marked cells.

Figure 6a is a FLIM tile patched from Fig. 1c, Fig. 6c is the corresponding virtual H&E image, and Fig. 6b is the true H&E image as the reference. An experienced pathologist annotated 7 different cells commonly found in the TME on virtual histology image and confirmed the true one, including tumour, fibroblasts, lymphocytes, plasma cells, neutrophils, macrophages, and RBC. Afterwards, lifetime histograms are derived for all the cells annotated, and averaged lifetime were extracted. Figure 6d clearly demonstrates the lifetime differences of the cells. Interestingly, the averaged lifetimes for various cell types follow a progressive increase: RBCs have an average lifetime of 0.251 ns, fibroblasts 0.376 ns, tumours 0.424 ns, lymphocytes 0.471 ns, neutrophils 0.659 ns, plasma cells 0.69 ns, and macrophages have the longest average lifetime at 2.42 ns. RBC show the shortest lifetime and appear too dark in the FLIM image (depicted as dark blue dots in Fig. 6a) to be discerned. On the other hand, macrophages have the longest lifetime and are easily identifiable in the FLIM image (represented as large blue dots in Fig. 6a). Plasma cells and neutrophils possess similar lifetime values, resulting in their visual similarity (highlighted by green dots indicated by yellow and blue arrows in Fig. 6a). In comparison, lymphocytes are less visually distinguishable than these two cell types. Tumour cells and fibroblasts exhibit shorter lifetime values, making them appear dimmer in FLIM images compared to other cells. While most tumour cells can be identified in FLIM with the aid of the H&E image as a reference, pinpointing fibroblasts remains a challenging task on the FLIM image.

Figure 6d illustrates the distribution of lifetime of the cells. This variability across different cell types or even within the same cell type is anticipated, which arises from the diverse composition of molecules within individual cells, each with its own characteristic lifetime. In the context of Fig. 6, a specific lifetime suggests the presence of a specific molecule shared among all 7 cell types, with the counts on the y-axis reflecting the relative abundance of this molecule within the cells. In practice, distinguishing between different cell types often involves leveraging both intensity and lifetime data. While intensity provides valuable morphological information, lifetime measurements offer insights into molecular composition. By analysing cellular-level histograms of lifetime, users can gain a deeper understanding of the functional characteristics of various cell types, complementing the morphological details provided by intensity data.

### Comparison of different image formats

Various DL-based approaches have been proposed to leverage label-free autofluorescence images for the generation of virtual histological images, such as^17^. Since FLIM images also contain autofluorescence intensity, we will compare synthetic histology images generated from three different formats. These formats include autofluorescence intensity images in greyscale, false-colour lifetime images with normalised intensity as the alpha channel (*α*-FLIM)^27,28^, and intensity-weighted false-colour lifetime images (IW-FLIM). For intensity images, we followed the post-processing described in^17^. In the case of false-colour lifetime images, the process involves initially converting greyscale lifetime images into 3-channel RGB images through colour mapping, with a fixed range of [1.0 ns, 5 ns]. Subsequently, the normalised intensity is appended as the alpha channel to create *α*-FLIM, resulting in four-channel images. For the generation of IW-FLIM images, the normalised intensity is employed as a soft weight, undergoing pixel-wise multiplication with each channel in the false-colour lifetime images. Note that despite different processing methods, *α*-FLIM and IW-FLIM images are visually identical. These three formats were then inputted into the DL model with the same hyper-parameters for training on the same training dataset.

**Fig. 7:**
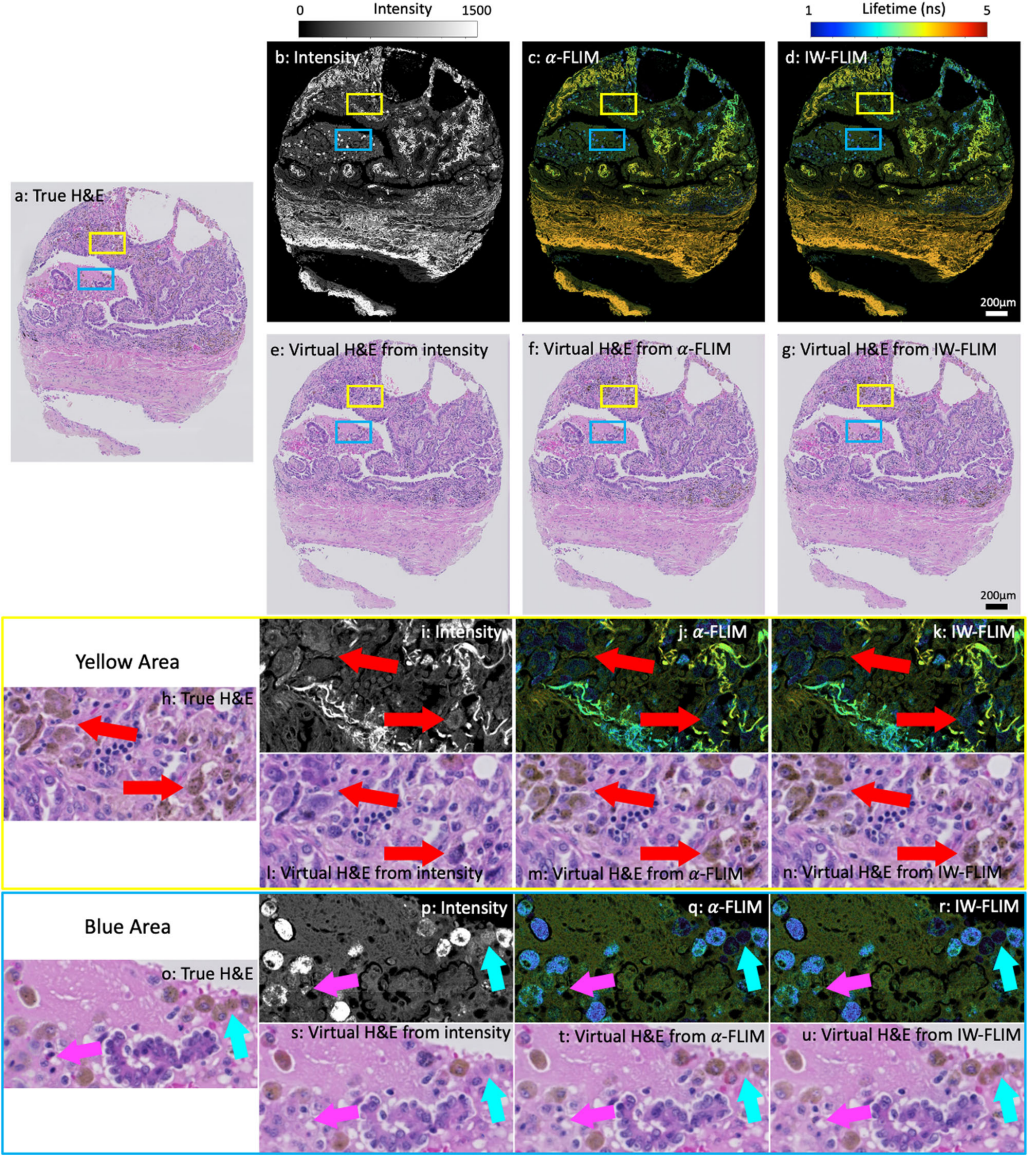
Comparison of virtual H& E staining using intensity, false-colour FLIM with the intensity image as the alpha channel (*α*-FLIM), and intensity-weighted false-colour FLIM (IW-FLIM). (**a**), (**h**), and (**o**) are the true H& E staining. (**e**), (**i**), and (**s**) are the virtual H& E staining from intensity (**b**), (**i**), and (**p**), respectively. (**f**), (**m**), and (**t**) are the virtual H& E staining from *α*-FLIM (**c**), (**j**), and (**q**), respectively. (**g**), (**n**), and (**u**) are the virtual H& E staining from IW-FLIM (**d**), (**k**), and (**r**), respectively. The yellow and blue rectangles highlight variations in the synthetic results. The red arrows point out macrophages that are entirely absent in the intensity-based synthesis (**i**) but appear in the *α*-FLIM (**j**) and IW-FLIM-based (**k**) outcomes. Synthesis based on intensity can reconstruct macrophages when the signal is strong (bright dots pointed out by the cyan arrows in (**p**)), but it struggles to identify those with dimmer signals (the grey dot surrounded by the bright dots, as indicated by the cyan arrow in **p**). The pink arrows also indicate a few missing immune cells in intensity-based synthesis.

Figure 7 depicts the synthesis of virtual H&E staining from the aforementioned three image formats. Visual inspection by three pathologists is carried out initially to assess the overall quality of the synthesis. This evaluation reveals that all three formats can generate satisfactory virtual H&E-stained images, without compromising the detection and diagnosis of lung cancer. However, some mis-reconstructed details are observed in intensity-based synthesis, which can impact the biological analysis of the tissue.

In our study, a noticeable limitation of intensity-based reconstruction is its inability to locate some macrophages. In Fig. 7, the red arrows point to clusters of macrophages that are misinterpreted as tumour cells in virtual H&E staining from intensity (Fig. 7i), whereas they are clearly visible in the virtual images generated from *α*-FLIM (Fig. 7j) and IW-FLIM (Fig. 7k). However, macrophages can be accurately identified in intensity images when they exhibit strong signals, as indicated by the bright dots highlighted by the blue and cyan arrows in the intensity images (Fig. 7s). Additionally, a few immune cells are misclassified as other cell types in the intensity-based virtual image, as shown by the pink arrow in Fig. 7s. In contrast, both *α*-FLIM (Fig. 7t) and IW-FLIM (Fig. 7u) have excellent capabilities for virtual H&E staining.

To quantify the comparison, we applied four predominant metrics^29^ for measuring the similarity between true and virtual H&E-stained images, including the normalised root mean-squared error (NRMSE), the normalised mutual information (NMI), the peak signal-to-noise ratio (PSNR), and the mean structural similarity index metric (NSSIM). The results are listed in Table 1 and the best one is highlighted in bold for each metric. Overall, the quantitative results are consistent with the visual results shown in Fig. 7, where all values are comparable. Intensity images are less effective than false-colour FLIM images for synthesising virtual images, with IW-FLIM being the most suitable format for achieving optimal virtual H&E staining. *α*-FLIM also produces competitive results similar to IW-FLIM, with marginal differences compared to IW-FLIM.

**Table 1:** Quantitative comparison of virtual H&E staining on two TMA sections of lung cancerous tissues from intensity, *α*-FLIM, and IW-FLIM using the normalised root-mean-squared error (NRMSE), the normalised mutual information (MNI), the peak signal-to-noise ratio (PSNR), and the mean structural similarity index metric (MSSIM).

		NRMSE	NMI	PSNR	MSSIM
Lung sample 1 (Fig. 1a)	Intensity	0.127	1.093	20.085	0.529
	a-FLIM	0.127	1.091	20.121	0.53
	IW-FLIM	**0.126**	**1.096**	**20.202**	**0.533**
Lung sample 2 (Fig. 1c)	Intensity	0.095	1.154	22.767	0.689
	a-FLIM	0.093	1.161	22.962	0.695
	IW-FLIM	**0.092**	**1.164**	**23.026**	**0.697**

Note that smaller NRMSE and NMI indicate more similar images, whereas larger PSNR and MSSIM represent better similarity between images.

## Discussion

This study described a method to generate clinical-grade virtual H&E staining from label-free FLIM images acquired on unstained tissue samples, with the assistance of the pix2pix network^19^ and the DISTS loss^25^. Despite a variety of virtual histological staining methodologies having been proposed, our study differs from existing methods in several aspects. Our FLIM images were obtained using a confocal microscope at the optimal excitation and emission wavelengths determined by a *λ*-to-*λ*/spectral scan on the tissue provided by the Leica FLIM system, containing the intensity and the corresponding lifetime images. Although Borhani et al. utilised lifetime images in their study, their input images were collected using a multi-modality microscope, resulting in a combination of 16-channel spectral intensity and single-channel lifetime^20^.

Using FLIM for virtual H&E staining yields superior results, compared to existing methods, such as autofluorescence intensity images, which is primarily attributed to the additional information introduced by fluorescence lifetime. An initial scan of the unstained tissue using a commercial FLIM imaging system can identify the optimal excitation/emission wavelengths best suited for the samples, ensuring the acquisition of high-quality FLIM images. Since FLIM contains both intensity and lifetime information, our previous work and this study demonstrate that combining both information can achieve optimal outcomes^30^. In addition, the section “ Comparison of different image formats” indicated that intensity-weighted false-colour lifetime images are most suited for the virtual staining. Consequently, our method applies intensity-weighted false-colour images as the input into a supervised GAN model for paired image-to-image translation.

Despite the prevalence of FLIM in biomedical and clinical applications, most FLIM image analysis methods primarily rely on statistical techniques, such as histogramming, to summarise the data. These approaches are effective at idenjpgying dominant components within FLIM images but may fall short when it comes to providing cellular-level information. This limitation becomes particularly apparent when attempting to discern the intricate details of cellular components within complex environments like the tumour microenvironment (TME). This may be overcome by co-registering FLIM with histology images^10^, which is still challenging, and sometimes may be infeasible. Virtual H&E staining can address the limitations by offering a rapid and precise reference for FLIM images at the cellular level. This reference enables the identification of distinct lifetime signatures associated with various cell types within tissue samples, as illustrated in Fig. 6. By providing this valuable reference instantly, virtual H&E staining greatly expands the potential applications of FLIM beyond its current scope. This advancement has great potential for label-free characterisation of both morphological and phenotypical features of cells, opening up new possibilities for research and clinical purposes.

Compared to other imaging modalities used in the literature for label-free virtual H&E staining, our FLIM microscope is more complicated and usually requires longer time to image tissue samples. For example, the images presented in Fig. 1, 3, 4, and Supplementary Fig. 1a–d were acquired within 40 min per TMA core with a pixel size of 455 nm. However, following the optimisation of scanning parameters, the scanning time reduced to less than 15 min while maintaining the same quality. This allows us to scan a TMA core within 30 min at a much higher resolution (a pixel size of 200 nm), as shown in Supplementary Fig. 1e–j. On the other hand, H&E stain is a routine test in biology and pathology laboratories, which takes about 30 min for the process. It seems counterproductive to use FLIM for virtual H&E staining, given the time on FLIM imaging. Considering the quality of FLIM-based virtual H&E staining, we believe it is worthwhile. Additionally, FLIM has demonstrated the capability for providing metabolic information, without requiring exogenous biomarkers^1,4–7,31^. All these together have great potential to address challenges in managing small biopsies and limited tissue samples in advanced-stage lung cancer patients undergoing only small biopsies rather than resection procedures. By leveraging FLIM, we can optimise tissue utilisation by reducing the necessity for additional ancillary tests while achieving the same level of diagnostic and phenotypic characterisation.

In the process of sample preparation and data acquisition, a common practice for virtual histological staining from autofluorescence images involves first scanning unstained samples using autofluorescence imaging modalities and subsequently staining the same samples^17,18,32^. This approach offers a significant advantage in achieving optimal co-registration at the pixel level with minimal distortion on both images, which is crucial for the effective utilisation of supervised DL techniques. To achieve this, diverse techniques have been applied, such as multi-stage elastic-based registration^17^ or RANSAC-based warping^20^. Nonetheless, our study suggests that with the same sample preparation and data collection procedure, employing an affine transformation for the co-registration is adequate to achieve satisfactory virtual staining, thus, substantially reduce technical uncertainties for enhanced reproducibility. Supplementary Fig. 2 illustrates that the synthetic results with additional elastic registration do not contribute to better virtual staining, which may attribute to the sample preparation described in the section “Sample preparation and data collection”.

It is important to note that although we leveraged pix2pix and DISTS as our DL method, we believe that other advanced models could also be exploited for the purpose. Indeed, many models have proved to be effective for virtual H&E staining, such as UNet-based GAN^17,32,33^, FCNN-p2p and VGG-a2p^20^, or conditional GAN^18^. Generally, all DL models described in^34^ for image-to-image translation may be applicable to DL-based virtual histological staining. However, direct applications may not be feasible for this purpose. We experimented this with three advanced DL models without any optimisation, including the original pix2pix GAN, ResVit^35^, and Denoising diffusion GAN (DDGAN,^36^), and the results are depicted in Supplementary Fig. 3. Despite the success of these models, they are unable to generate acceptable virtual H&E staining. Therefore, incorporating advanced loss functions to further refine the learning process would also be beneficial for achieving optimal results. For example, the perceptual loss^37^ can be employed for high-level feature reconstruction while preserving realistic textural information, and the texture loss^38^ can help retain fine textural details from the original images. The DISTS loss^25^ harnessed in this study combines spatial information with textural features, making it a more favourable choice for enhancing the reconstruction.

Various virtual histological staining techniques have been proposed over the past few years^14^, and our approach perfectly complements these methods in a novel aspect. Existing methods for virtual H&E staining usually facilitate autofluorescence images acquired at specific excitation/emission wavelengths using specific filters, such as DAPI and Cy5 channels^17^. In contrast, our FLIM image acquisition utilised excitation and emission wavelengths determined through a *λ*-to-*λ* scan of the lung tissue samples, resulting in optimal excitation (485 nm) and emission ([500 nm, 720 nm]) wavelengths for our study. This represents a fundamental difference in our approach compared to other proposals, which is independent of specific pre-set filters, allowing us to collect high-quality images at optimal excitation/emission wavelengths. This enhancement was confirmed through blinded evaluations by experienced pathologists and quantitative assessments using commonly used similarity metrics (see the section “Comparison of different image formats”).

## Methods

### Sample preparation and data collection

Surgically resected, early stage non-small cell lung carcinomas were identified from the clinical archival and a TMA was constructed as previously described^39^. FLIM images were collected using a confocal FLIM system (Leica STELLARIS 8 FALCON FLIM Microscope) with an 20x/0.75NA objective. The excitation and emission wavelengths were set at 485 nm and [500 nm, 720 nm], which was determined by a *λ*-to-*λ* scan of the tissue. The image size was fixed at 512 × 512 pixels with various pixel sizes. Following FLIM acquisition the same sample underwent rehydration in a series of ethanol dilutions, staining with Haematoxylin and Eosin and then dehydration and subsequent imaging on a bright-field slide scanner (ZEISS Axio Scan.Z1), with an 20x/0.75NA objective. The digitalised H&E-stained images have a pixel size of 0.22 *μ*m. In total, 84 lung cancer tissue samples (including 69 TMA cores scanned with the pixel size 0.455 *μ*m, 5 biopsies scanned with the pixel size at 0.4 *μ*m, and another 10 TMA cores from a separate cohort scanned with the pixel size at 0.25 *μ*m), 4 slides of colorectal cancer scanned with the pixel size 0.4 *μ*m, and four sections of endometrial cancer scanned with the pixel size 0.4 *μ*m were facilitated for this study.

### Data post-processing

**Fig. 8:**
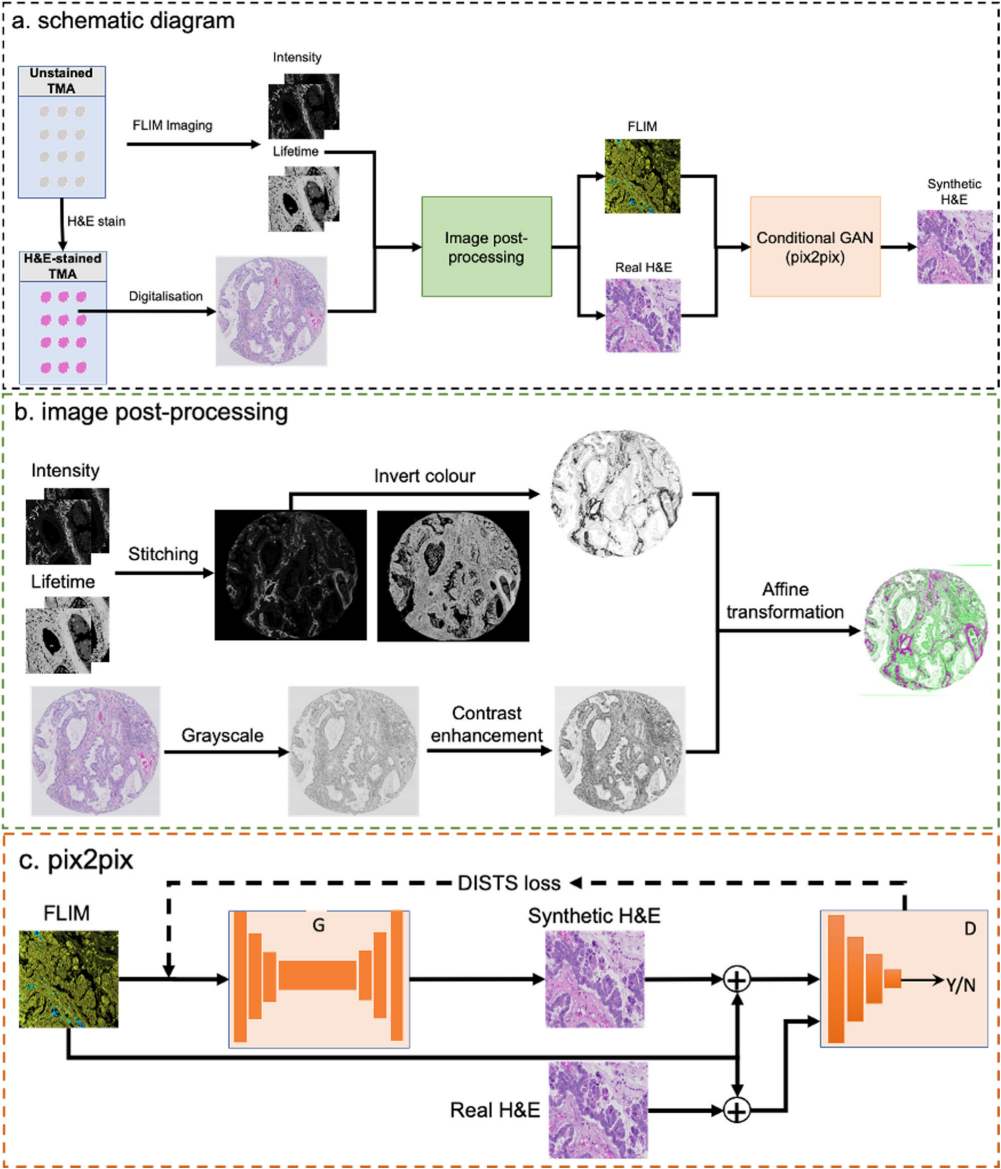
Schematic diagram of the proposed method. (**a**) is the overall procedure of the method. (**b**) is the image post-processing to generate paired FLIM and H& E images. (**c**) is the conditional GAN (pix2pix) and DISTS loss for the synthesis.

FLIM images were reconstructed by an exponential fitting on photon histogram counts to estimate lifetimes using the Leica LAX-X software and exported as intensity and lifetime images separately. Note that to ensure the consistency when visualising FLIM images, raw intensity data is limited to [0, 2000] and raw lifetime is to [0.0 ns, 5.0 ns]. Afterwards, all tiles per core within the tumour microarray (TMA) were stitched using MIST^40^. To achieve optimal registration, only intensity images were exploited. Similar to refs. ^17^, intensity images were colour inverted for the white background. H&E images were converted to greyscale and contrast-enhanced by saturating 1% of the values at both low and high bounds of the intensity range. The processed intensity and H&E images were used as the input for the co-registration. Since the tissue processing did not change the underlying structural and cellular information, Affine transformation in MATLAB was employed for the co-registration of FLIM and H&E images (Fig. 8b).

The co-registered images are then resampled to the patches of 256 × 256 pixels, where those patches with over 75% background were disregarded. For the input into the pix2pix network, three different formats were tested. The first is the intensity images, the second is the false-colour lifetime images with the corresponding normalised intensity images as the alpha channel, and the third is the false-colour lifetime images pixel-multiplied with the corresponding intensity images. Note that intensity images are globally normalised per TMA core before applying to the lifetime images.

### GAN Architecture

In this study, the pix2pix network^19^ was utilised and a conceptual diagram is depicted in Fig. 8c. Essentially, the pix2pix GAN is a conditional model, where the generator is U-Net-like architecture and the discriminator is a multi-layer classifier using the input FLIM images as the condition. The objective of the discriminator is given by:

1 $${{{\mathcal{L}}}}\left(G,D\right)={{\mathbb{E}}}_{f,s}\left[\log D\left(s| \,f\right)\right]+{{\mathbb{E}}}_{f,h}\left[\log \left(1-D\left(h| f\right)\right)\right]$$

where *f*, *s*, and *h* are FLIM, synthetic H&E, and true H&E images, respectively, and ${\mathbb{E}}$ is the expectation. The objective of the generator is:

2 $${{{\mathcal{L}}}}\left(G\right)={{\mathbb{E}}}_{f,s,h}\left[{\left\vert \left\vert h-G\left(f,s\right)\right\vert \right\vert }_{1}\right]$$

where *L*_1_ distance is applied. Accordingly, the overall objective for the GAN is:

3 $${{{\mathcal{L}}}}=a{{{\mathcal{L}}}}\left(G,D\right)+\beta {{{\mathcal{L}}}}\left(G\right)$$

where *α* and *β* are the regularisation parameter to adjust the weight of the discriminator and generator, respectively.

### DISTS Loss

The regularisation terms in the original GAN networks are usually insufficient to synthesise acceptable virtual histological staining and various extra constraints have been applied to overcome the challenges, such as total variations (TV)^17,32,41^ and Structural Similarity Index Measure (SSIM)^20,42^. To achieve the optimal synthesis, the Deep Image Structure and Texture Similarity (DISTS) metric is employed in this study, which yields superiority to existing single metrics by combining structural and texture information together and has proven to be tolerant to mild local and global structural distortion^25^.

Given *r* and *s* are true and synthetic images, respectively, and ${{{\mathcal{F}}}}$ is a function to retrieve feature maps of input images from a trained VGG model, the extracted features ${\tilde{x}}_{j}^{(i)}$ can be defined as ${{{\mathcal{F}}}}(x)=\{{\tilde{x}}_{j}^{(i)};i\,=\,\in [0,5];j\in [1,{n}_{i}];x\in [r,s]\}$, where ${\tilde{x}}^{(0)}=x,i$ is *i*th convolution layer, and *n*_*i*_ is feature maps in the *i*th convolution layer. DISTS is defined as:

4 $${{{{\mathcal{L}}}}}_{DISTS}(r,s,\eta ,\theta )=1-\mathop{\sum }\limits_{i=0}^{m}\mathop{\sum }\limits_{j=1}^{{n}_{i}}\left({\eta }_{ij}{{{\mathcal{T}}}}({{{\mathcal{F}}}}(r),{{{\mathcal{F}}}}(s))+{\theta }_{ij}{{{\mathcal{S}}}}({{{\mathcal{F}}}}(r),{{{\mathcal{F}}}}(s))\right)$$

where *η* and *θ* are learnable weights, satisfying $\mathop{\sum }\nolimits_{i = 0}^{m}\mathop{\sum }\nolimits_{j = 0}^{{n}_{i}}({\eta }_{i,j}+{\theta }_{i,j})=0,{{{\mathcal{T}}}}$ and ${{{\mathcal{S}}}}$ are quality measurement for the texture and structure, respectively, governed by:

5 $${{{\mathcal{T}}}}({{{\mathcal{F}}}}(r),{{{\mathcal{F}}}}(s))=\frac{2{\mu }_{{\tilde{r}}_{j}}^{(i)}{\mu }_{{\tilde{s}}_{j}}^{(i)}+{c}_{1}}{{\left({\mu }_{{\tilde{r}}_{j}}^{(i)}\right)}^{2}+{\left({\mu }_{{\tilde{s}}_{j}}^{(i)}\right)}^{2}+{c}_{1}}$$

and

6 $${{{\mathcal{S}}}}({{{\mathcal{F}}}}(r),{{{\mathcal{F}}}}(s))=\frac{2{\sigma }_{{\tilde{r}}_{j}\,{\tilde{s}}_{j}}^{(i)}+{c}_{2}}{{\left({\sigma }_{{\tilde{r}}_{j}}^{(i)}\right)}^{2}+{\left({\sigma }_{{\tilde{s}}_{j}}^{(i)}\right)}^{2}+{c}_{2}}$$

where ${\mu }_{{\tilde{r}}_{j}}^{(i)},{\mu }_{{\tilde{s}}_{j}}^{(i)},{\sigma }_{{\tilde{r}}_{j}}^{(i)}$, and ${\sigma }_{{\tilde{s}}_{j}}^{(i)}$ are the global means and variances of the extracted feature maps for *r* and *s*, respectively, ${\sigma }_{{\tilde{r}}_{j}\,{\tilde{s}}_{j}}^{(i)}$ is the global covariance between the feature maps, and *c*_1_ and *c*_2_ are tiny values for numeric stability.

Combining Eq. (3) and Eq. (4), the final objective of the network is:

7 $${{{\mathcal{L}}}}=a{{{\mathcal{L}}}}\left(G,D\right)+\beta {{{\mathcal{L}}}}\left(G\right)+\lambda {{{{\mathcal{L}}}}}_{DISTS}(r,s,\eta ,\theta )$$

where *λ* is another regularisation parameter for DISTS loss.

### Blinded evaluation of virtual and true H&E staining

A set of 12 lung TMA cores were facilitated for the blinded evaluation, where all images were anonymised to allow three experienced pathologists to perform unbiased inspection. To holistically appraise the quality of the images, pathologists meticulously examined each image’s four fundamental aspects: nuclei clarity via Haematoxylin staining, cytoplasm precision via Eosin staining, overall staining integrity, and suitability for diagnostic decision-making. For each parameter, pathologists judiciously assigned scores aligned with established criteria, ranging from Unacceptable (1) to Acceptable (2), Excellent (3), and Exemplary (4).

### Implementation details

The model was implemented using PyTorch and adapted for three input formats, including greyscale intensity images, false-colour lifetime images with normalised intensity as the alpha channel, and false-colour lifetime images pixelwise weighted by normalised intensity. Adaptive Moment Estimation optimiser^43^ was deployed with *β*_1_ as 0.5 and *β*_2_ as 0.999. Learning rate was initialised at 0.0001 and decayed by 10 at very 60 epochs. Total epochs were set to 300. *α*, *β*, and *λ* in Eq. (7) were set to 0.1, 1, and 5, respectively.

72 lung cancer samples (including 70 TMA cores and 2 biopsies) were utilised for the training and 15 independent lung cancer sections (including 12 TMA cores and 3 biopsies) were split out as the independent testing set. For colorectal and endometrial cancers, both were trained via transfer learning on 2 sections and evaluated on two separate sections. The patches in the training stage were fixed at 256 × 256. Simple data augmentations, including horizontal flipping and 15-degree rotation, were applied during the training. Training was performed on NVIDIA V100 GPUs provided by the EPSRC Tier-2 National HPC Services Cirrus hosted by EPCC (https://www.epcc.ed.ac.uk/hpc-services/cirrus) at The University of Edinburgh.

### Supplementary information

Supplementary Information

## Data Availability

All data generated or analysed during this study are included in this published article.

## Code availability

The pix2pix used in this study is publicly available at https://github.com/mrzhu-cool/pix2pix-pytorch. The DISTS loss is available at https://github.com/dingkeyan93/DISTS. FLIM images were stitched using Fiji MIST stitching plugin (https://github.com/usnistgov/MIST). MATLAB was used for affine transformation to co-register FLIM and true histology images.
