# Author Correction: Deep learning-based virtual H& E staining from label-free autofluorescence lifetime images

**DOI:** 10.1038/s44303-025-00115-w

**Published:** 2025-10-14

**Authors:** Qiang Wang, Ahsan R. Akram, David A. Dorward, Sophie Talas, Basil Monks, Chee Thum, James R. Hopgood, Malihe Javidi, Marta Vallejo

**Affiliations:** 1 Centre for Inflammation Research, Institute of Regeneration and Repair, The University of Edinburgh, Edinburgh, UK; 2 Translational Healthcare Technologies Group, Centre for Inflammation Research, Institute of Regeneration and Repair, The University of Edinburgh, Edinburgh, UK; 3 Department of Pathology, Royal Infirmary of Edinburgh, Edinburgh, UK; 4 School of Engineering, The University of Edinburgh, Edinburgh, UK; 5 School of Mathematical and Computer Sciences, Heriot-Watt University, Edinburgh, UK; 6 Department of Computer Engineering, Quchan University of Technology, Quchan, Iran

**Keywords:** Engineering, Cancer imaging

Correction to: *npj Imaging* 10.1038/s44303-024-00021-7, published online 28 June 2024

In this article the ethics and informed consent statement was missing and should have been as follows: “This study complied with all relevant ethical regulations. The TMA study was approved by the NHS Lothian NRS Bioresource (REC No. 20/ES/0061), with a specific application SR1949. Written informed consent was not required as only archival samples were used, which was approved by delegated authority granted to R&D by the NHS Lothian Caldicott Guardian (Application number CRD19031).”. The original article has been corrected.

