# Supplementary material for: Deep learning-based virtual H& E staining from label-free autofluorescence lifetime images
Source: Npj Imaging. 2024 Jun 28;2:17. doi: 10.1038/s44303-024-00021-7 (PMC11213708; doi:10.1038/s44303-024-00021-7)
Supplement: Supplementary file 1 — Supplementary Information [file 44303_2024_21_MOESM1_ESM.pdf]

Supplementary information for "Deep learning-based virtual H&E staining from label-free autofluorescence lifetime images"

Qiang Wang[1,2*], Ahsan R Akram[1,2], David A Dorward[1,3], Sophie Talas[1,3], Basil Monks[3], Chee Thum[3], James R Hopgood[4], Malihe Javidi[5,6], Marta Vallejo[5]

[1]Centre for Inflammation Research, Institute of Regeneration and Repair, The University of Edinburgh, Edinburgh, UK.

[2]Translational Healthcare Technologies Group, Centre for Inflammation Research, Institute of Regeneration and Repair, The University of Edinburgh, Edinburgh, UK.

[3]Department of Pathology, Royal Infirmary of Edinburgh, Edinburgh, UK.

[4]School of Engineering, The University of Edinburgh, Edinburgh, UK.

[5]School of Mathematical and Computer Sciences, Heriot-Watt University, Edinburgh, UK.

[6]Department of Computer Engineering, Quchan University of Technology, Quchan, Iran.

Table of Contents

Supplementary Fig. 1. Virtual H&E staining from FLIM conducted using the same cohort as depicted in Fig. 1..... 3

Supplementary Fig. 1 (cont.). Virtual H&E staining from FLIM conducted on a separate cohort. 4

Supplementary Fig. 1 (cont.). Virtual H&E staining from FLIM conducted on a separate cohort as depicted in Supplementary Fig.1e-h. 5

Supplementary Table 1: Blind evaluation of six lung cancer tissues by three pathologists. 6

Supplementary Table 1 (cont.): Blind evaluation of six lung cancer tissues by three pathologists. 7

Supplementary Fig. 2. Comparison of Virtual H&E with affine and elastic registration. 8

Supplementary Fig. 3. Virtual H&E staining by various DL models. 9

Supplementary Fig. 4. Virtual H&E staining with random noise. 10

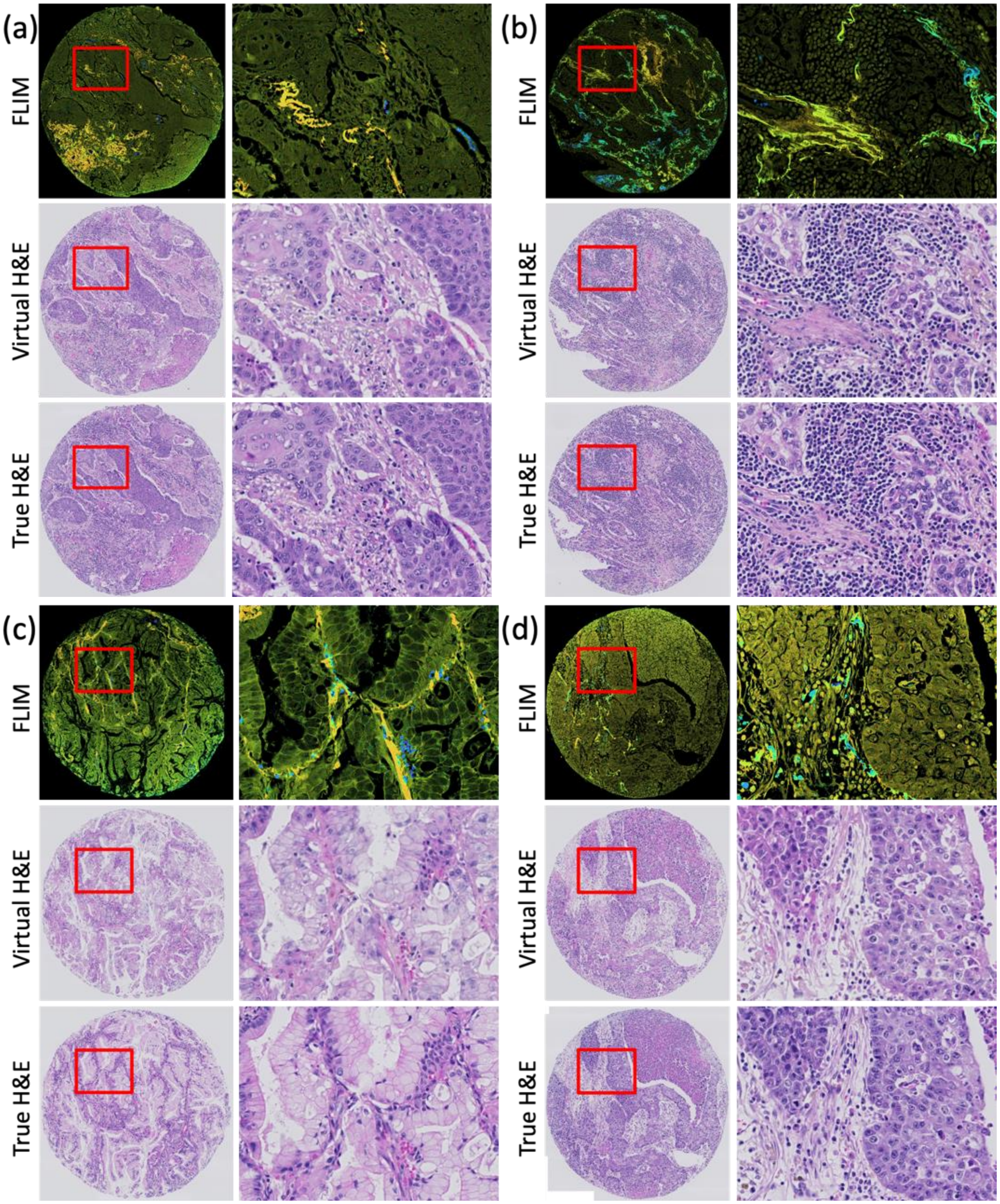

Supplementary Fig. 1. Virtual H&E staining from FLIM conducted using the same cohort as depicted in Fig. 1.

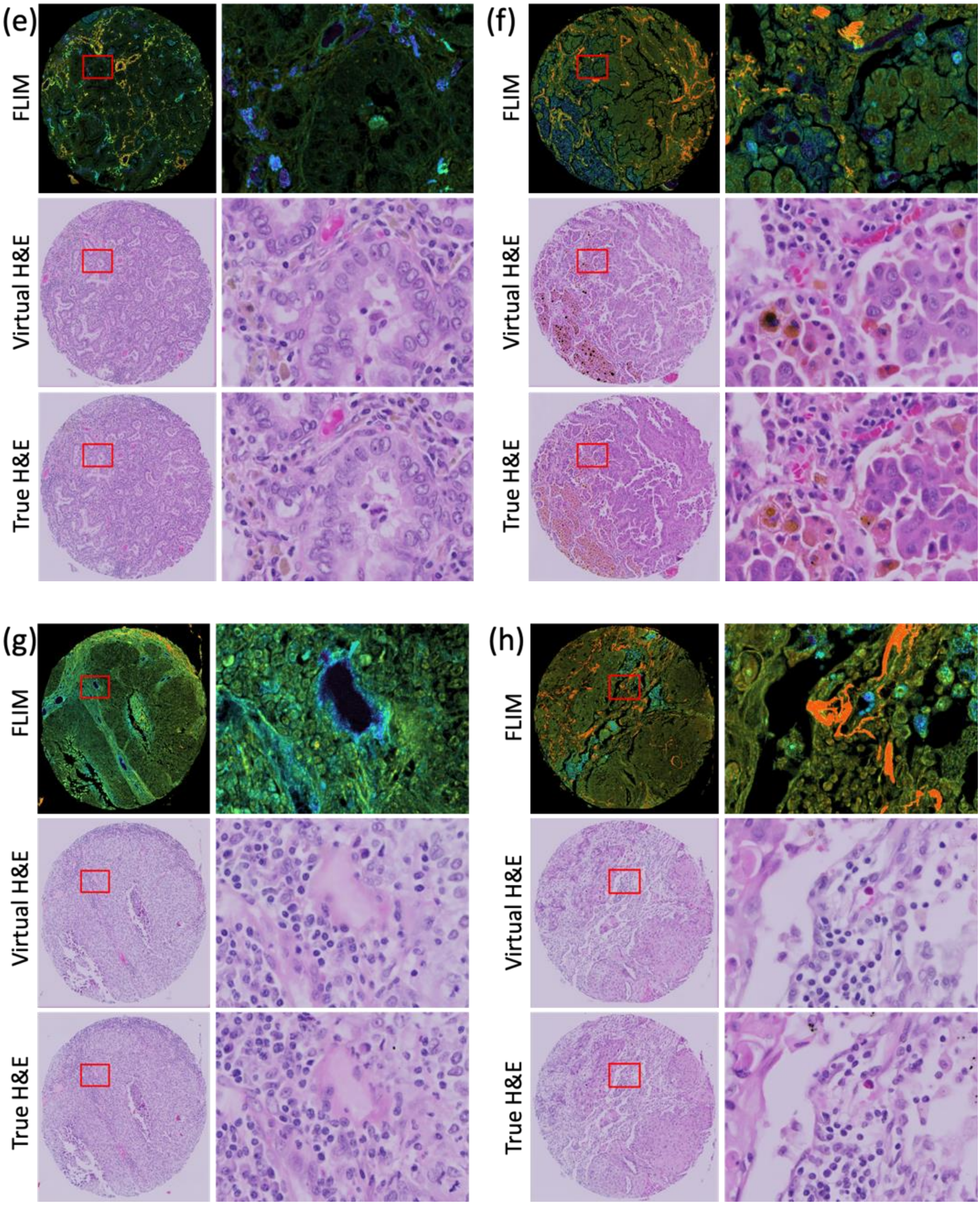

Supplementary Fig. 1 (cont.). Virtual H&E staining from FLIM conducted on a separate cohort.

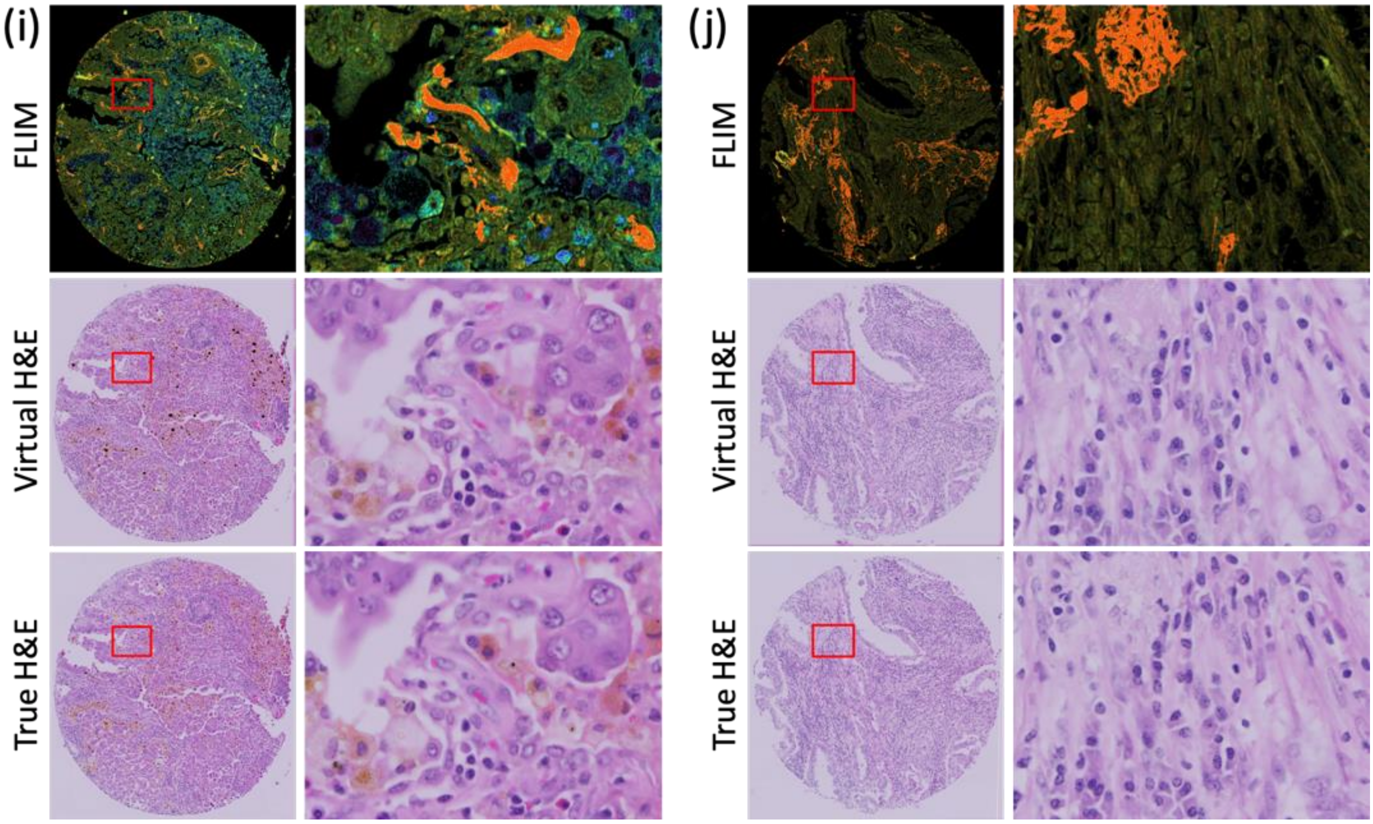

Supplementary Fig. 1 (cont.). Virtual H&E staining from FLIM conducted on a separate cohort as depicted in Supplementary Fig.1e-h.

Supplementary Table 1: Blind evaluation of six lung cancer tissues by three pathologists. The evaluation includes nuclei detail, cytoplasm detail, overall stain quality, and diagnosis decisions, each with a score of 1 (unacceptable), 2 (acceptable), 3 (excellent), and 4 (perfect).

	**Pathologists**		**Scores**				**Average**
		Types	Nuclei	Cytoplasm	Overall	Diagnosis	
Sample 1	Pathologist 1	Real	4	4	4	4	4
		Virtual	4	4	4	4	4
	Pathologist 2	Real	4	3	3	4	3.5
		Virtual	3	3	3	3	3
	Pathologist 3	Real	3	3	3	3	3
		Virtual	3	3	3	3	3
Sample 2	Pathologist 1	Real	4	4	4	4	4
		Virtual	3	3	3	3	3
	Pathologist 2	Real	4	4	4	4	4
		Virtual	4	4	4	4	4
	Pathologist 3	Real	3	2	2	2	2.25
		Virtual	2	2	2	2	2
Sample 3	Pathologist 1	Real	4	4	4	4	4
		Virtual	3	3	3	3	3
	Pathologist 2	Real	3	3	3	4	3.25
		Virtual	3	4	3	4	3.5
	Pathologist 3	Real	3	3	2	3	2.75
		Virtual	2	2	2	2	2
Sample 4	Pathologist 1	Real	4	4	4	4	4
		Virtual	4	4	4	4	4
	Pathologist 2	Real	3	3	3	3	3
		Virtual	3	3	3	4	3.25
	Pathologist 3	Real	3	3	3	3	3
		Virtual	3	3	3	3	3
Sample 5	Pathologist 1	Real	3	3	3	3	3
		Virtual	3	3	3	3	3
	Pathologist 2	Real	2	2	2	3	2.25
		Virtual	2	2	2	3	2.25
	Pathologist 3	Real	3	2	3	3	2.75
		Virtual	3	2	2	2	2.25
Sample 6	Pathologist 1	Real	3	3	3	3	3
		Virtual	4	4	4	4	4
	Pathologist 2	Real	2	2	2	3	2.25
		Virtual	3	3	3	3	3
	Pathologist 3	Real	2	2	2	2	2
		Virtual	3	2	2	2	2.25
Sample 7	Pathologist 1	Real	4	4	4	4	4
		Virtual	4	4	4	4	4
	Pathologist 2	Real	3	3	4	4	3.5
		Virtual	3	3	3	4	3.25
	Pathologist 3	Real	2	2	2	2	2
		Virtual	2	2	2	2	2

Supplementary Table 1 (cont.): Blind evaluation of six lung cancer tissues by three pathologists. The evaluation includes nuclei detail, cytoplasm detail, overall stain quality, and diagnosis decisions, each with a score of 1 (unacceptable), 2 (acceptable), 3 (excellent), and 4 (perfect).

	**Pathologists**		**Scores**				**Average**
		Types	Nuclei	Cytoplasm	Overall	Diagnosis	
Sample 8	Pathologist 1	Real	4	3	4	4	3.75
		Virtual	3	3	3	3	3
	Pathologist 2	Real	3	3	3	3	3
		Virtual	3	3	3	3	3
	Pathologist 3	Real	3	2	2	2	2.25
		Virtual	2	1	2	2	1.75
Sample 9	Pathologist 1	Real	4	3	4	4	3.75
		Virtual	3	3	3	3	3
	Pathologist 2	Real	3	3	3	3	3
		Virtual	3	3	3	3	3
	Pathologist 3	Real	3	3	3	3	3
		Virtual	3	2	2	2	2.25
Sample 10	Pathologist 1	Real	4	4	4	4	4
		Virtual	3	4	3	4	3.5
	Pathologist 2	Real	3	3	3	3	3
		Virtual	3	3	3	3	3
	Pathologist 3	Real	3	3	2	3	2.75
		Virtual	2	2	2	2	2
Sample 11	Pathologist 1	Real	4	4	4	4	4
		Virtual	4	4	4	4	4
	Pathologist 2	Real	4	4	4	4	4
		Virtual	4	4	4	4	4
	Pathologist 3	Real	2	2	2	2	2
		Virtual	3	3	3	3	3
Sample 12	Pathologist 1	Real	4	4	4	4	4
		Virtual	4	4	4	4	4
	Pathologist 2	Real	3	3	3	3	3
		Virtual	3	2	2	3	2.5
	Pathologist 3	Real	2	2	1	2	1.75
		Virtual	2	2	2	2	2

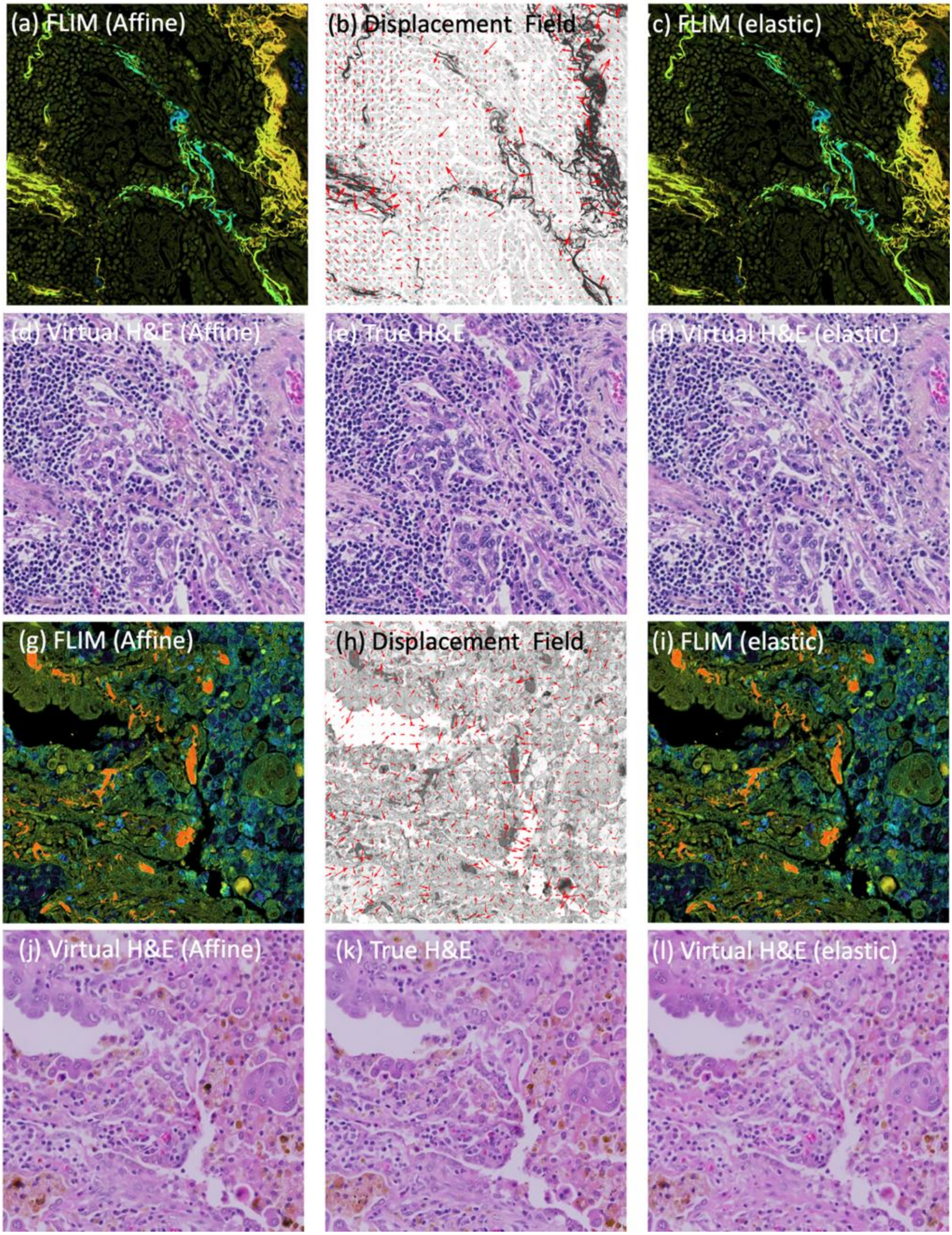

Supplementary Fig. 2. Comparison of Virtual H&E with affine and elastic registration. Two FLIM images, after affine transformation (a and g), undergo further transformation using elastic registration (c and i). The corresponding displacement fields are presented in b and h, respectively. Notably, for optimal illustration, the vectors in b and h have been scaled up 10 times as the original vectors are too small. Virtual H&E images of a, c, g, and i are shown in d, f, j, and l, respectively. It is evident that additional elastic registration does not lead to further improvement in virtual H&E staining.

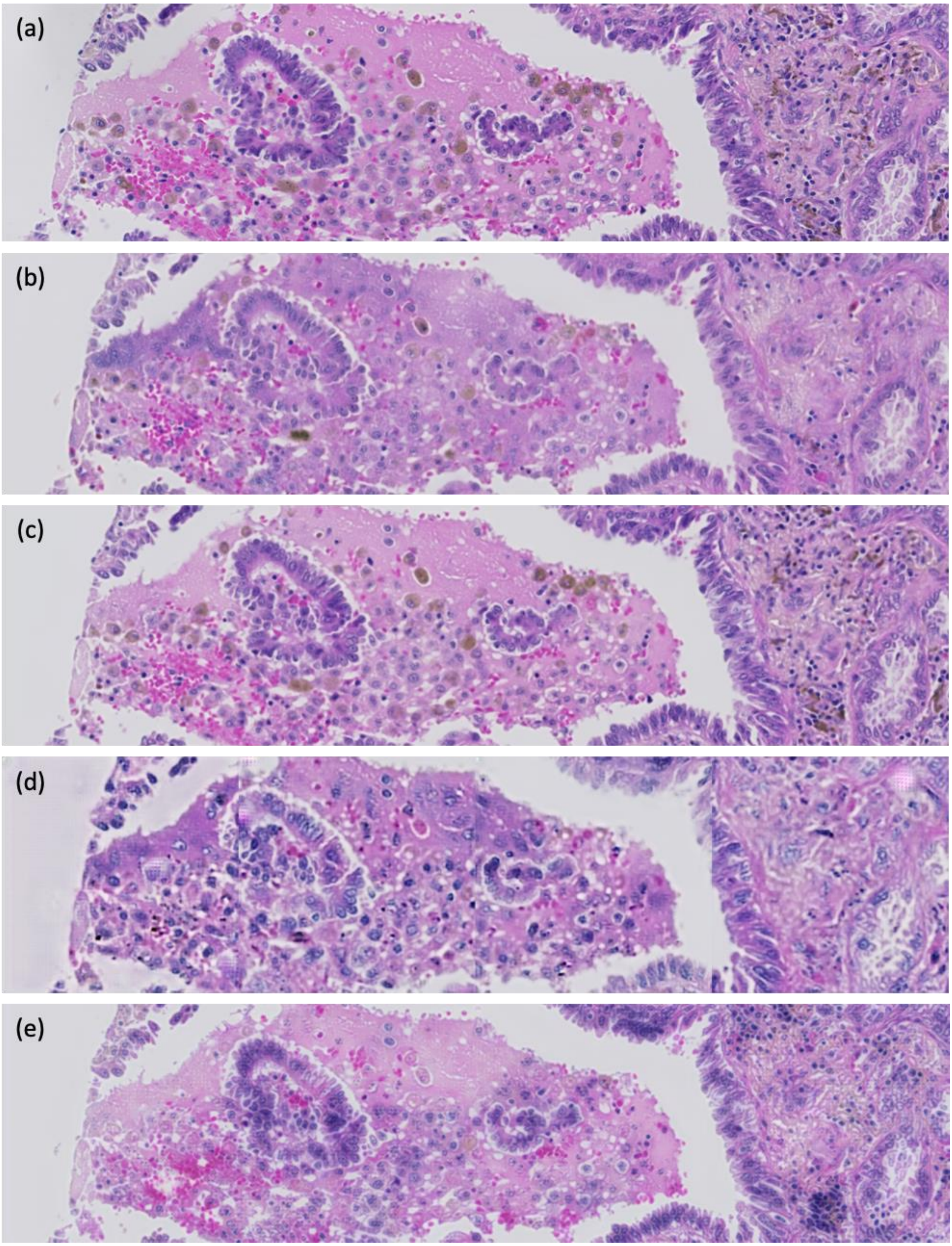

Supplementary Fig. 3. Virtual H&E staining by various DL models. (a) true H&E staining. (b) pix2pix without additional loss functions. (c) pix2pix with DISTS loss. (d) the original ResVit [1]. (e) the original DDGAN [2]. Without any further optimisation, pix2pix, ResVit and DDGAN cannot generate satisfactory virtual H&E staining.

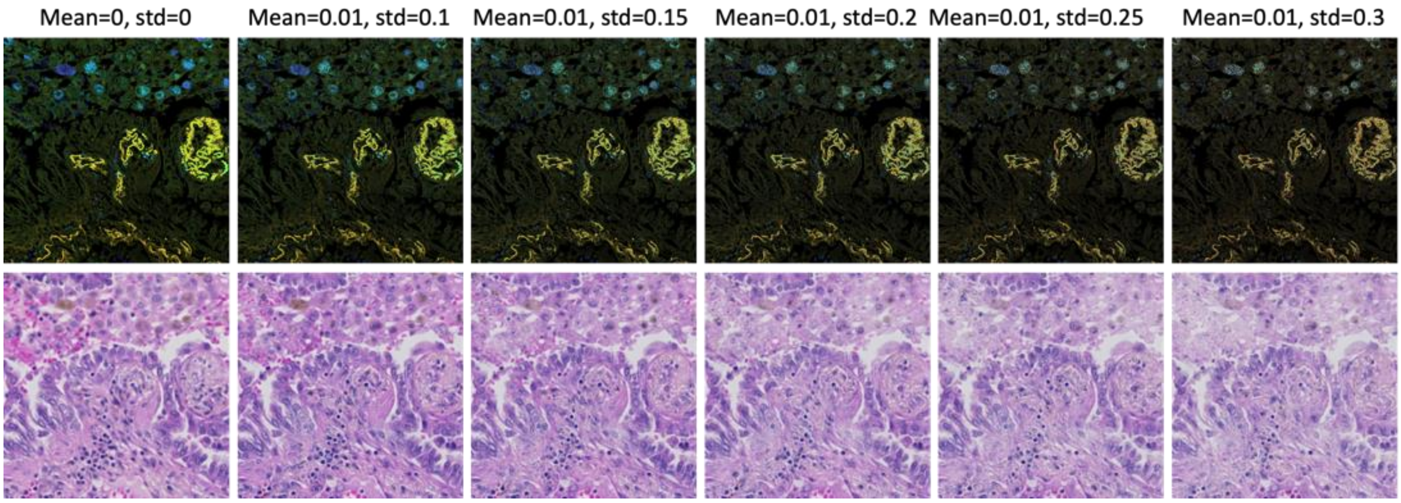

Supplementary Fig. 4. Virtual H&E staining with random noise. The noise was sampled using Gaussian distributions with a mean of 0.01 and standard deviations ranging from 0.1 to 0.3. With a fixed mean value, it is evident that increasing the standard deviation significantly compromises the quality of the reconstruction.

References

1. Dalmaz, O., Yurt, M., & Çukur, T. (2022). ResViT: Residual vision transformers for multimodal medical image synthesis. *IEEE Transactions on Medical Imaging*, *41*(10), 2598-2614.
2. Xiao, Z., Kreis, K., & Vahdat, A. (2021). Tackling the generative learning trilemma with denoising diffusion gans. *arXiv preprint arXiv:2112.07804*.
